# Pathways to Financial Success: An Empirical Examination of Perceived Financial Well-Being Based on Financial Coping Behaviors

**DOI:** 10.3389/fpsyg.2021.762772

**Published:** 2021-11-01

**Authors:** Junguo Shi, Saif Ullah, Xun Zhu, Shanshan Dou, Faiza Siddiqui

**Affiliations:** ^1^Institute of Industrial Economics, Jiangsu University, Zhenjiang, China; ^2^School of Finance and Economics, Jiangsu University, Zhenjiang, China; ^3^School of Business Administration, National College of Business Administration and Economics, Lahore, Pakistan; ^4^School of Management, Jiangsu University, Zhenjiang, China; ^5^Program in Sustainability Management, Inha University, Incheon, South Korea

**Keywords:** financial well-being, financial coping behaviors, financial socialization, financial satisfaction, early childhood experiences

## Abstract

The current study empirically tests a financial well-being (FWB) model built on financial socialization (FS) and early childhood consumer experience (ECCE). The current study was conducted based on primary data obtained through structured questionnaires. By using a convenient sampling technique, data were collected from 1,500 respondents from Pakistan. Results advocated that childhood experiences directly affect the FWB in adults. In addition, FS agents, such as parents, have direct and indirect effects on the FWB in adults. Findings revealed that financial coping behaviors mediate the relationship among FS agents, such as parents, students, and the FWB. Surprisingly, FS agent peers do not impact adult FWBs. The study concluded that FWB could be improved by socializing with parents and teachers and using childhood experiences. Considering the importance of the role of parents and teachers, they should discuss financial issues with children. Policymakers should work to provide some opportunities for children so that they can practice and gain experience.

## Introduction

Improving the quality of life has been an area of interest among social scientists for the last few decades, which can be improved by improving well-being (Brüggen et al., 2017; Cherney et al., 2019; Nguyen-Cousin et al., 2019; Sabri and Anthony, 2019; Sabri et al., 2020). Well-being is also essential as it helps overcome health, family, and interpersonal problems (McLellan, 2017). Irrespective of the significance of well-being, researchers debate over the definition, measure, and factors affecting well-being (Rea, 2017). Several studies argued that well-being is a combination of psychological, social, financial, and physical well-being (Zemtsov and Osipova, 2016). However, financial well-being (FWB) is the most essential and fundamental factor that explains adults' well-being (Netemeyer et al., 2018). Individuals' FWB is a relatively new concept in the field of personal financial management. Although an escalating body of literature regarded FWB, no agreement exists over the definition, measure, and factors affecting FWB (Rea, 2017). For instance, Shim et al. (2009b) explained FWB as “overall satisfaction with own financial situation.” However, Drever et al. (2015) concluded that FWB is the capacity to handle regular and periodic finances, the ability to cope with financial uncertainties, the accomplishment of financial targets, and the liberty to make choices that enable one to enjoy life. In other words, Drever et al. (2015) explained FWB as proficiencies in managing financials, whereas Shim et al. (2009b) defined FWB as an outcome of financial management skills. FWB can be objective and subjective. Shim et al. (2009b) supported the overall satisfaction as an indicator of subjective FWB, whereas Danes and Yang (2014) illuminated objective FWB as income adequacy and net worth. Following Shim et al. (2009b), the present study considered FWB as subjective, that is, satisfaction with one's financial situation.

Throughout the lives of adults, FWB plays a key role as it affects physical, psychological, and social well-being, resulting in poor work performance, loss of attention, reduced productivity, and higher truancy. Many adolescents now experience significant challenges to their FWB although many look forward to overcoming these hazards and reaching FWB but cannot do so. In the era of the COVID-19 pandemic, achieving FWB becomes more difficult (Azizi et al., 2021). Although the success and failure in achieving FWB depend on the resources and opportunities, some activities will enrich FWB across individuals. Through better future planning and effective use of resources, these behaviors promote sound financial decisions. The predominant literature suggested that these habits are guided by a wide variety of socio-economic characteristics and a complex set of personal traits (Gutter and Copur, 2011; Sabri, 2011; Danes and Yang, 2014; Strömbäck et al., 2017; Fan et al., 2018; Netemeyer et al., 2018; Sabri et al., 2020).

Various contributing factors have been established in the studies to examine FWB, that is, competence in financial knowledge and skills (Kim and Chatterjee, 2013; Xiao et al., 2013; Hazudin et al., 2018; Russell et al., 2020); physical health, marital status, and income (Cox et al., 2009; Netemeyer et al., 2018; Islam and Schreyer, 2020); interaction with parents and attitudes toward knowledge (Vosloo, 2014; Furnham and Cheng, 2017; Mohamed, 2017; Netemeyer et al., 2018); interaction with socialization agents (Sabri, 2011; Kim and Chatterjee, 2013; Rea, 2017; Rea et al., 2019; Lanz et al., 2020) and early childhood consumer experiences (ECCEs) (Brüggen et al., 2017; Netemeyer et al., 2018; Rea et al., 2019); positive financial behaviors (Serido et al., 2010; Farrell et al., 2016; Prawitz and Cohart, 2016; Furnham and Cheng, 2017; Sabri et al., 2020); and parent–child communication and expectations of parents (Serido et al., 2010; Drever et al., 2015; Aquilino and Supple, 2016; Serido and Deenanath, 2016). The present study attempts to find the relevant factors contributing to an individual's FWB in Pakistan.

Financial socialization (FS) can affect the FWB. FS is the acquisition and evolution of beliefs, capabilities, aspirations, standards, behaviors, and activities facilitating individual well-being and financial viability. Although FS is essential to understand the financial behavior among young adults, only a few scholars studied the FS process from young adults' perspectives. Given the lack of empirical and qualitative data, understanding of these behaviors is often overlooked (Rea, 2017). Previous studies indicated that children learn from their families about FS processes at younger ages, thereby impacting their potential financial activities and FWB (Shim et al., 2009a; Gudmunson and Danes, 2011; Kim and Chatterjee, 2013; Danes and Yang, 2014; Gudmunson et al., 2015; Rea et al., 2019). Based on the literature, we claim that FS contributes toward FWB.

Danes and Yang (2014) indicated that when adults mature and grow their own families, they try to practice the financial behavior patterns they learned as a child. The recent literature on consumer socialization suggested that individuals' behaviors and knowledge in adulthood were learned during childhood through the influence of different socialization agents (Sabri, 2011; Kim and Chatterjee, 2013; Legenzova et al., 2019; Rea et al., 2019). Moschis and Churchill (1978) claimed four primary FS agents, namely, parents, peers, education, and media. However, Gudmunson et al. (2016), Gutter et al. (2010), Hastings et al. (2013), Kim and Chatterjee (2013) argued that parents, peers, and teachers are the most important FS agents. Following the relevant literature, in the present study, we have included parents, peers, and teachers as socialization agents.

Empirical studies on the FWB demonstrated the significance of ECCE in explaining FWB. ECCE has a lasting effect on the financial actions of adults (Garg and Singh, 2018). Individuals in their adulthood practice the financial behaviors that they have learned in their childhood. Danes and Yang (2014) argued that, as adults grow their own families, they try to follow the financial behavior patterns they learned as a child. These learned financial behaviors help adults to achieve financial confidence. The results of developmental studies showed that ECCE positively affects adult financial management abilities (Kim and Chatterjee, 2013). The outcome of these financial management skills is FWB. In the present study, we consider ECCE's role in explaining adults' FWB.

The previous literature documented the effect of FS through parents (FSP), peers (FSPE), and teachers (FST), and ECCEs on FWB. However, along with these factors, the proper management of finances requires some life skills (Drever et al., 2015). These life skills help individuals to transform abilities and experiences into behaviors. To achieve FWB, individuals should have a wide range of skills (Danes and Yang, 2014). By following this, we can say that Adults develop life skills through FS and ECCE that help individuals achieve FWB. In the present study, we have only considered financial coping behaviors (FCB) (Serido et al., 2010; Stein et al., 2012; Britt et al., 2016) as life skills.

Coping behaviors (CBs) are strategies that are taken in response to problems. Coping is an ongoing process of adaptation in life. In addition, coping can be a reactive coping to deal with current problems and prevent future problems or proactive copying to attain future goals (Schwarzer and Knoll, 2003; Greenglass and Fiksenbaum, 2009). We can apply the concept of CBs in personal finance. Consistent with the literature, the three types of FCB are as follows: (a) reactive CBs to cope with instantaneous change in financial situations (e.g., paying credit card bills by using another card, cutting expenses), (b) preventive behaviors to control future financial problems (e.g., spending within budget, paying off credit card balances each month), and (c) proactive CBs to arrange for future goals (e.g., saving money) (Serido et al., 2010, 2014; Hamilton et al., 2018). Serido et al. (2010) argued that only future-oriented CBs are relevant to FWB.

The FCB observed in adulthood results from the financial behaviors learned in childhood through the interaction with the socialization agents and experiences (Serido et al., 2014). FCB helps individuals to lower financial stein and achieve financial success. Serido et al. (2010) concluded that FCB mediates the relationship between FS and financial well-being. Consistent with the personal financial literature (Xiao, 2008; Serido et al., 2010, 2014), in this study, we have only considered future-oriented CBs that are preventive FCB (budgeting and spending within the budget) and proactive FCB (saving and investing). We expect FS and ECCE to affect the FWB and CBs that help explain this relationship. In other words, FCB will mediate the relationship among FS, ECCE, and FWB.

This research aims to add to our current knowledge of personal attributes and FS that promote the FWB of an individual. To do so, we propose a model of FS and ECCE on FCB and their connection with FWB. Although developmental studies examined these factors (Sabri, 2011; Drever et al., 2015; Netemeyer et al., 2018; Rea et al., 2019; Lanz et al., 2020; Mubeen et al., 2020; Sabri et al., 2020; Ullah and Yusheng, 2020), they applied other combination of variables to study FWB.

The current study focuses on Pakistani adults. Pakistan is one of the world's six most populated countries. Pakistan is also among the countries with the fastest-growing populations. Adults make up the majority of Pakistan's population (~60% of the population is over 30 years old). However, the country cannot gain from this youth because of political instability, bad financial conditions, inadequate health and educational systems, rising unemployment rates, and a high population growth rate. According to a UNDP study released recently, Pakistan is rated 152nd out of 189 nations in the Human Development Index. Unemployed people are more likely to suffer from mental health issues, such as depression, than employed ones. All these affect one's quality of life, and one method to improve quality of life is to increase adults' FWB. The government of Pakistan is also trying to improve the quality of life by improving the well-being of adults (Mamirkulova et al., 2020; Wang et al., 2021). Thus, the current study is important for Pakistan as it will provide information on aspects connected to individuals' FWB, which would improve their quality of life.

## Theoretical Background and Hypothesis

### FS

FS is the study and development of beliefs, knowledge, norms, standards, attitudes, and behaviors that enhance financial viability and individual well-being (Danes, 1994). The establishment of standards, values, norms, and attitudes that will either hinder or support the development of financial capacity among individuals and enhance FWB is all part of FS. Although attitude formation is a growing field of study in psychology, few studies focused on developing financial attitudes in children. People's financial activities in adulthood result from financial views that they learned as children (Drever et al., 2015). Abbas et al. (2019a) showed that students' enrollment has a positive effect on their learning abilities that result in better financial decisions in adulthoods.

People learn about consumer knowledge and behaviors in their childhood through interactions with socialization agents, such as parents, teachers, other family members, classmates, religion, and schools, and they put this information and behavior into effect as adults (Gudmunson and Danes, 2011; Gutter and Copur, 2011; Drever et al., 2015). Many of the FS outcomes (e.g., good financial behaviors, FWB) of young people are ingrained in the FS processes experienced in childhood (e.g., financial literacy) (Shim et al., 2009b; Drever et al., 2015; Gudmunson et al., 2015). Jorgensen et al. (2016) recently investigated the influence of FS on financial decision-making and found that people who have more opportunity to interact with and witness FS agents make better financial judgments. Rea et al. (2019) also verified the findings of the FS theory that financially socialized people make better financial decisions and attain FWB in adulthood. In the present study, the role of parents, peers, and teachers as a socialization agents is considered.

The study proposes the following hypotheses:

H1: In young adults, FS affects FWB.
H1a: The FWB of young adults is influenced by their parents' FS.
H1b: Peers' FS has an impact on young adults' FWB.
H1c: Teachers play an important role in understanding adult FWB.

### ECCE

The socialization process begins in childhood and continues to some extent throughout one's life (Danes, 1994). Baltes (1987) presented the notion of the life span, which provides a lens to analyze the consistency and variance in behavior throughout a person's life. According to the idea of life span, distinct developmental activities may become important for a time at different phases of an individual's life span. When people reach adulthood, these activities are influenced by social and economic circumstances, thereby become more difficult. They also create the foundations for later actions. According to this hypothesis, early consumer encounters have a substantial influence on people's financial behavior.

Danes and Yang (2014) also pointed out that once children grow up and start their own families, they strive to emulate the financial behavior patterns they learned in childhood. According to Sabri and MacDonald (2010), students who received ECCE were more likely to participate in active financial behavior and were less likely to report financial issues (Sabri and MacDonald, 2010). Falahati and Sabri (2015) found that early consumer experiences are important predictors of adult financial distress in their study. As a result, we may conclude that early consumer experiences aid in the development of smart financial judgments and the management of financial stress, which enhances adults' FWB.

The study then proposes the second hypothesis:

H2: ECCE affects individuals' FWB.

### Mediating Role of FCB

CBs are actions done in reaction to difficulties. Coping is a life-long process of adaptability, which can be reactive coping to deal with present issues, proactive copying to achieve future objectives, or preventative coping to avoid future issues (Schwarzer and Knoll, 2003; Greenglass and Fiksenbaum, 2009). The notion of CBs may be applied to personal finance. According to the literature, FCB have three types: (a) reactive CBs to deal with immediate changes in financial situations (e.g., paying credit card bills with another card, cutting expenses), (b) preventive CBs to deal with future financial problems (e.g., sticking to a budget, paying off credit card balances each month), and (c) proactive CBs to deal with long-term financial problems (Serido et al., 2010, 2014; Hamilton et al., 2018).

CBs are most commonly researched in the literature as a reactive stress reaction when resources are limited. From another aspect, Greenglass (2002) proposed a new theory of coping, The Proactive Coping Theory of Coping, which states that proactive CB aims at accomplishing future-oriented goals. Greenglass and Fiksenbaum (2009) stated that proactive CB has an impact on overall well-being. According to Serido et al. (2010), only proactive CB future-oriented coping strategies are important to financial habits. They also claimed that financial coping strategies developed in infancy as a result of interactions with socialization agents, and experiences are carried over into adulthood. According to Helm et al. (2019), proactive financial CB has a beneficial effect on psychological well-being. Following these studies, the present study evaluated future-oriented CB: proactive financial CB (budgeting and spending within the budget) and preventative financial CB (budgeting and spending within the budget) (saving and investing).

By mediating the relationship between resources and output, proactive CB helps to explain the resources–output paradigm, according to the proactive coping hypothesis. Greenglass (2002) utilized proactive CB as a mediating variable to explain the influence of FS on the FWB by viewing FS as psychological resources and well-being as output. Serido et al. (2010) also looked at the function of CBs in explaining the relationship between FS and well-being and concluded that financial coping practices moderate the relationship between FWB and FS. Although proactive CB's mediating function was indicated two decades ago, the investigation uncovered just one study by Serido et al. (2010) who investigated the mediating role of CB.

Serido et al. (2010) regarded FSP but neglected socialization through teachers and peers. They also overlooked the impact of consumer experiences on FWB. The current study aims to close this gap. We expect ECCEs and FS to have an influence on FWB and financial coping strategies, which will help us explain this link. In other words, the link among FS, ECCEs, and FWB will be mediated by FCB.

The third and fourth hypotheses of the study are as follows:

H_3_: FCB mediates the relationship between FS and FWB.
H_3a_: The FCB mediates the relationship between FSP and FWB.
H_3b_: The FCB mediates the relationship between FSPE and FWB.
H_3c_: FCB mediates the relationship between FST and FWB.
H_4_: FCB mediates the relationship between ECCE and FWB.

### Theoretical Framework

**Figure 1:**
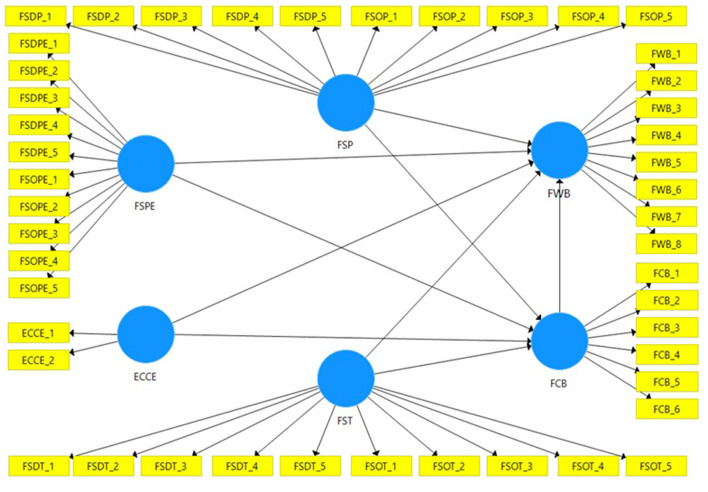
Theoretical framework. Source: Researcher.

Figure 1 depicts the theoretical framework used in this investigation, which was based on relevant literature and assumptions. Figure 1 indicates that, in addition to ECCE, FSP, FSPE, and FST is also an independent variable. The dependent variable is FWB. The locus of control hypothesis attempts to explain the link among FS agents, ECCEs, and FWB. The yellow boxes display the objects used to measure the variables, whereas the blue cycles show the variables.

## Materials and Methods

This section comprises different parts, including the following: (a) population, sampling, and collection of data, (b) questionnaire design, and (c) statistical model.

### Population, Sampling, and Collection of Data

This research focuses on a population of up to 40 years of age belonging to Pakistan. Respondents involved people that were younger than 40 years of age and engaged in some earning practice. Typically, before going into the 1940s, citizens strive to establish long-lasting and enduring ties with other people, finalize their careers, select their professions, and most notably, start to handle their families and lives. The researchers applied this condition to assess the exact situation of the FWB. The unit of analysis is individuals. The researcher applied the sampling technique because data collection from the whole population is time-consuming, so sampling is a good choice for the researcher (Vanderstoep and Johnston, 2009). In this study, the researcher applied the convenient sampling technique. Under this technique, a subset of the conveniently available population is used for data collection.

The questionnaire was in English. Although English is not a primary language in Pakistan, it is a means of education in the country. The citizens of Pakistan can therefore read and comprehend the English language. Thus, the researchers used the study questionnaire in the English language. Researchers obtained data from respondents who have been able to understand the English language. Data were gathered by visiting the various communal media and large companies. Before answering the questionnaire, researchers briefed the respondents on the study goal. After the briefing, the researchers circulated the study surveys to the readily available respondents. During the data collection process, researchers gathered data from various geographical areas and sought to preserve diversity in gender, educational qualifications, and other critical indicators. Throughout the data collection process, the investigator maintained the anonymity of the respondents and adopted the moral procedures prescribed by the APA and the researcher's community. The resulting segment presents the demographic profile of the respondents. For a population of 1 million, Krejice and Morgan (1970) suggested a sample of 384. Cohen (1992) proposed minimum sample size of 212 for mediation analysis. However, by evaluating the sample size of similar studies (Sabri, 2011; Serido et al., 2014; Burcher et al., 2018; Sabri and Anthony, 2019; Abbas et al., 2020; Paulson et al., 2021), a sample size of 1,500 is considered acceptable for this analysis. The researcher used a large sample size to generalize the findings of the investigation.

### Measures

The present research was cross-sectional and validated tools used to collect data. Important variables examined include FWB, FCB, FS, and ECCE.

#### FWB

The researchers assessed FWB using the “In-charge Financial Distress/Financial Well-Being Scale (IFDWF)” created by Prawitz et al. (2006). This instrument measures the level of stress, and well-being resulting from one's financial conditions. The response varied between 1 and 10 for each item, ranging from 1 (lowest FWB) to 10 after reversing the average individual rating (highest FWB).

#### FS

In the current study, the researcher assessed FS using the Financial Social Learning Opportunities (FSLO) method produced by the researcher (Gutter et al., 2010; Gudmunson and Danes, 2011). The FSLO score was a composite measure based on six dimensions: “discussions with parents,” “discussions with peers,” “discussions with teachers,” “observation of parents,” “observation of peers,” and “observation of teachers.” The average FS score ranged from 1 (lowest FS) to 5 (highest FS).

#### ECCE

The researcher has assessed ECCE using the tool developed by Sabri (2011). Sample questions were “at what age they became involved in financial activities, which included (a) having their own savings account, and (b) discussing financial matters with parents.” The researchers measured ECCE by taking the average of the above two questions. The average score was from 1 (low ECCE level) to 4 (higher ECCE level).

#### FCB

The researchers measured individuals' responses toward preventive and proactive FCB using a six-item scale developed by Serido et al. (2010). All six items are measured on a five-point Likert scale. A score equal to 1 shows the lowest tendency toward FCB, whereas 5 shows the highest FCB.

### Procedure for Statistical Data Analysis

Following procedures for the data analysis were used.

#### Preliminary Data Analysis

The researcher applied the Preliminary Data Analysis to check missing values and outliers in the data. The data's missing values occur when respondents do not respond to all the voluntary or involuntary items. Missing values are not important; their patterns are important (Dong and Peng, 2013). Missing values create a problem in the results' generalizability, increasing standard errors and decreasing statistical power. The researchers have checked the missing values, where the missing values presented in the data were computed through linear interpolation. Outliers are cases with extreme values. Normally, outliers arise when the respondent is not a member of the selected population. Similar to missing values, outliers can also deteriorate the results. In the present study, the researcher has used box plots to detect outliers. Moreover, the researcher confirms that no outliers exist in the data.

#### Demographic Profile of the Respondents

The respondents' demographic profile helps the researchers specify the specific population group that was studied for the research purpose. The analysis of the demographics helps readers understand the results by keeping in mind the characteristics of that specific population. The respondents' demographic profile is not a direct part of the study; it only helps understand the results. Table 1 shows the results of the demographic profile of the respondents.

**Table 1:** Demographic profile of the respondents.

	**Frequency**	**Percent**		**Frequency**	**Percent**
**Age**			**Father's education**		
<25 years	328	29%	No Formal Education	96	8.5%
25–30 years	456	40.4%	High School and Lower	398	35.2%
30–40 years	346	30.6%	Undergraduate	202	17.9%
**Gender**			Bachelor	270	23.9%
Female	314	27.8%	Master	148	13.1%
Male	816	72.2%	Ph.D. degree	16	1.4%
**Marital status**			**Mother's education**		
Single	684	60.5%	No Formal Education	230	20.4%
Married	432	38.2%	High School and Lower	456	40.4%
Divorced	14	1.2%	Undergraduate	190	16.8%
**Education**			Bachelor	176	15.6%
High School and Lower	4	0.4%	Master	76	6.7%
Undergraduate	38	3.4%	Ph.D. degree	2	0.2%
Bachelor	148	13.1%	**Working experience**		
Master	824	72.9%	<3 years	498	44.1%
Ph.D. degree	116	10.3%	3–10 years	474	41.9%
**Academic ability**			Over 10 years	158	14.0%
GPA > 2.50	50	4.4%	**Place of origin**		
GPA 2.5–3.00	244	21.6%	Urban	750	66.4%
GPA 3.00–3.50	464	41.1%	Rural	380	33.6%
GPA <3.50	372	32.9%			

#### Descriptive Analysis of the Study Variables

Descriptive analysis of the study variables includes descriptive statistics and normality analysis of the study variables. Descriptive statistics include the study variables' minimum, maximum, mean, standard deviation, skewness, and kurtosis values. These values help to understand the basic nature of the variables. Normality analysis for the study variables is also performed. The parametric test is the assumption to check for the normality of the constructs. Normality analysis is performed by analyzing the skewness and kurtosis values. Table 2 shows the results of the descriptive statistics.

**Table 2:** Descriptive statistics.

	**Minimum**	**Maximum**	**Mean**	**Std. deviation**	**Skewness**	**Kurtosis**
FWB	1	10	4.97	1.93	0.21	(0.30)
FCB	1	4.83	2.38	0.66	1	0.30
FSP	1	5	3.29	0.83	(0.36)	(0.29)
FST	1	5	2.65	1.19	0.04	(1.16)
FSPE	1	5	2.98	0.99	(0.21)	(0.70)
ECCE	1	4	2.57	0.75	(0.25)	(0.38)

#### Evaluation of the Measurement Model

To test the measurement model, researchers need to assess the reliability and validity (in terms of content, convergence, and discriminant) (Hair et al., 2017).

#### Structural Model Assessment

The path modeling technique of partial least square (PLS) is used to evaluate the theoretical model. The method of PLS path modeling is similar to structural equation modeling (SEM). A special variant of SEM-based on variance is the PLS path modeling technique, whereas conventional SEM is a covariance-based technique. As both approaches have unique applications, these methods cannot be used alternatively. When the goal is to test a hypothesis where all relations between variables are predefined, SEM is preferred, whereas the PLS modeling technique is used when discovering new connections that are not predefined (Hair et al., 2017). In management sciences and related fields, PLS path modeling techniques are used (Hair et al., 2017). The sole objective of the current study was to determine key drivers of FWB among adults that can be adequately achieved by using Smartpls.

In addition, for studies with similar objectives, SEM is considered the most appropriate technique (Hair et al., 2017). We used SMART PLS software to compute the PLS results. Final decisions on the relationships among variables are made throughout the PLS path modeling technique based on their coefficients and t values.

#### Sobel Test

To compute the results related to the significance of the mediation effect, the Sobel test is used. Hair et al. (2017) recommended using the Sobel test when variables change their signs with and without the mediating variable. For the computation of the Sobel test, the methodology proposed by Preacher and Leonardelli (2001) is used. The results of the Sobel test are reported under the evaluation of the structural model. The Sobel test is widely used but lacks statistical power (Hair et al., 2017). Thus, in the present study, another test variance accounted for (VAF) is also applied.

#### VAF

VAF measures the size of the direct and indirect effect and, based on these measures, conclude about the mediation's presence. For the VAF, the indirect effect is considered, and if the indirect effect is between 20 and 80%, then partial mediation exists. However, if the VAF is above 80%, then full mediation exists. The results of VAF are also reported under the structural model assessment.

## Results

A total of 1,500 responses have been gathered by the researchers. Only 1,130 legitimate replies were left after removing the responses with missing values, unengaged responses, and outliers. Table 1 shows the demographics of the 1,130 respondents. The findings of the research revealed that the majority of the respondents (72%) were men between the ages 25 and 30 (40%). Furthermore, the bulk of the respondents were single (60%), had a master's degree (73%), and lived in metropolitan regions of the nation. Furthermore, ~85% of the population had fewer than 10 years of work experience.

Table 2 reports that the minimum value for FWB is one, and the maximum value is 10, with a mean value of 4.97. The respondents' mean value shows that adults are not financially satisfied with their current position nor are they in financial distress. The minimum and maximum values for the FCB are 1 and 4.83, respectively. A value of 1 shows the lowest FCB level, whereas a value of 4.83 shows the highest level of FCB. The mean value of the FCB is 2.38, which shows that less than the average respondents feel that they have CBs.

The minimum and maximum value of the FSP is 1 and 4.83, respectively. The mean value of FSP is 3.29, which shows that above-average respondents discuss and observe their parents' financial matters. The mean value also highlights that individuals mostly observe and discuss their financial matters with their parents as its average value is higher than the mean value of FSPE and FST. The minimum and maximum value for FSPE is 1 and 5, respectively. The mean value of the FSPE is 2.98, which shows that most of the respondents only sometimes observe and discuss their financial matters with their peers.

The minimum and maximum value of the FST is 1 and 5, respectively. The value of FST is 2.65, which shows that most respondents only sometimes discuss and observe their teachers' financial behaviors. However, the mean value of the teachers' FS is less than the mean value of FSP and FSPE, which indicates that most people observe and discuss their financial matters with their peers and parents.

According to Table 2, the minimum and maximum value for ECCEs is 1 and 4, respectively. A value equal to 1 shows minimum or no ECCEs, whereas 4 represents the respondents' highest level of childhood consumer experiences. The mean value of childhood consumer experiences is above the mean value of the scale, showing that most of the respondents have ECCEs.

Table 2 also reports the value of skewness and Kurtosis for the study variables. That is, all reported values of the skewness and Kurtosis are less than the absolute value of 2, which confirms that the skewness and kurtosis values are in the acceptable range (Field, 2017). Hence, the study variables have no problem with normality.

Hair et al. (2017) recommended that the researcher should validate data for normality and missing values before applying a path modeling technique. In section 3.3, the demographic profile of the respondents and descriptive analysis of the study variables are reported. Hair et al. (2017) proposed a two-step process to compute path modeling technique, namely, (1) evaluation of the measurement model and (2) evaluation of the structural model.

### Evaluation of the Measurement Model

The researchers checked the reliability, content, instrument, convergent, and instrument validity.

#### Instrument Reliability

The current study analyzed the reliability statistics by analyzing the outer factor loading of each construct individually (Hair et al., 2017). The loading factor above 0.4 is deemed appropriate (Field, 2017; Hair et al., 2017). Table 3 displays the external factor loading of each of the latent variables in the current study. Table 3 indicates that all performance values are significantly >0.4, suggesting that all current study variables follow the individual item reliability criterion.

**Table 3:** Factor loadings, average variance extracted, and composite reliability.

**Latent constructs and indicators**	**Standardized loadings**	**AVE**	**CR**	**Cronbach's alpha**
**Financial well-being**		0.57^a^	0.913	0.891
FWB_1	0.702			
FWB_2	0.77			
FWB_3	0.802			
FWB_4	0.808			
FWB_5	0.705			
FWB_6	0.692			
FWB_7	0.688			
FWB_8	0.854			
**Financial socialization—teachers**		0.708^a^	0.96	0.954
FSDT_1	0.849			
FSDT_2	0.806			
FSDT_3	0.861			
FSDT_4	0.863			
FSDT_5	0.823			
FSOT_1	0.841			
FSOT_2	0.831			
FSOT_3	0.867			
FSOT_4	0.863			
FSOT_5	0.805			
**Financial socialization—parents**		0.414^a^	0.875	0.847
FSDP_1	0.633			
FSDP_2	0.629			
FSDP_3	0.532			
FSDP_4	0.629			
FSDP_5	0.586			
FSOP_1	0.739			
FSOP_2	0.681			
FSOP_3	0.699			
FSOP_4	0.668			
FSOP_5	0.61			
**Financial socialization—peers**		0.573^a^	0.931	0.918
FSDPE_1	0.753			
FSDPE_2	0.714			
FSDPE_3	0.753			
FSDPE_4	0.813			
FSDPE_5	0.74			
FSOPE_1	0.721			
FSOPE_2	0.775			
FSOPE_3	0.747			
FSOPE_4	0.789			
FSOPE_5	0.76			
**Early childhood consumer experiences**		0.9^a^	0.947	0.89
ECCE_1	0.958			
ECCE_2	0.939			
**Financial coping behaviors**		0.481^a^	0.847	0.785
FCB_1	0.651			
FCB_2	0.637			
FCB_3	0.744			
FCB_4	0.788			
FCB_5	0.695			
FCB_6	0.633			

a *p < 0.05*.

#### Internal Consistency

Reliability of internal consistency measures how well an instrument calculates what the researcher wishes to use it to measure. Reliability is analyzed through a composite reliability coefficient, and a threshold value of 0.7 or higher is considered acceptable (Hair et al., 2017). Similarly, the reliability of the instruments is also reached by Cronbach's alpha (Field, 2017). Having a construct value of 0.6 or above is considered sufficient. Table 3 reports the coefficients of composite reliability of the study's latent variables. All values are above 0.7, indicating the composite accuracy of the study's tests. As shown in Table 3, Cronbach's alpha also shows that internal consistency exists in all measurements used in the present analysis.

#### Convergent Validity

Convergent validity or reliability of the predictor is the degree to which a measure is positively associated with the alternative means of the same construct (Hair et al., 2017). To test for convergent validity, average variance extracted (AVE) is used. To imply convergent validity, the AVE value should be at least 0.5 or more (Chin, 2010). Table 3 presents the derived average variance from all the constructs. All AVE values are above 0.5 except for parental financial coping patterns and FS. The AVE significance values imply that all values are statistically important, suggesting that the sample has ample convergent validity.

#### Validity of Discriminant

Validity of discriminant reveals the degree to which a given construct varies from other constructs. The Heterotrait-Monotrait (HTMT) ratio is used to test the validity of discriminants. The value of HTMT to suggest discriminant validity should be <0.9 (Henseler et al., 2014). All the HTMT values shown in Table 4 are <0.9; evidently, all research constructs are correct in discriminating terms.

**Table 4:** Discriminant validity [Heterotrait-monotrait ratio (HTMT)].

	**ECCE**	**FCB**	**FSP**	**FSPE**	**FST**	**FWB**
ECCE						
FCB	0.062					
FSP	0.201	0.367				
FSPE	0.134	0.286	0.651			
FST	0.125	0.313	0.626	0.713		
FWB	0.347	0.302	0.197	0.11	0.135	

### Structural Model Assessment (Hypothesis Testing)

**Figure 2:**
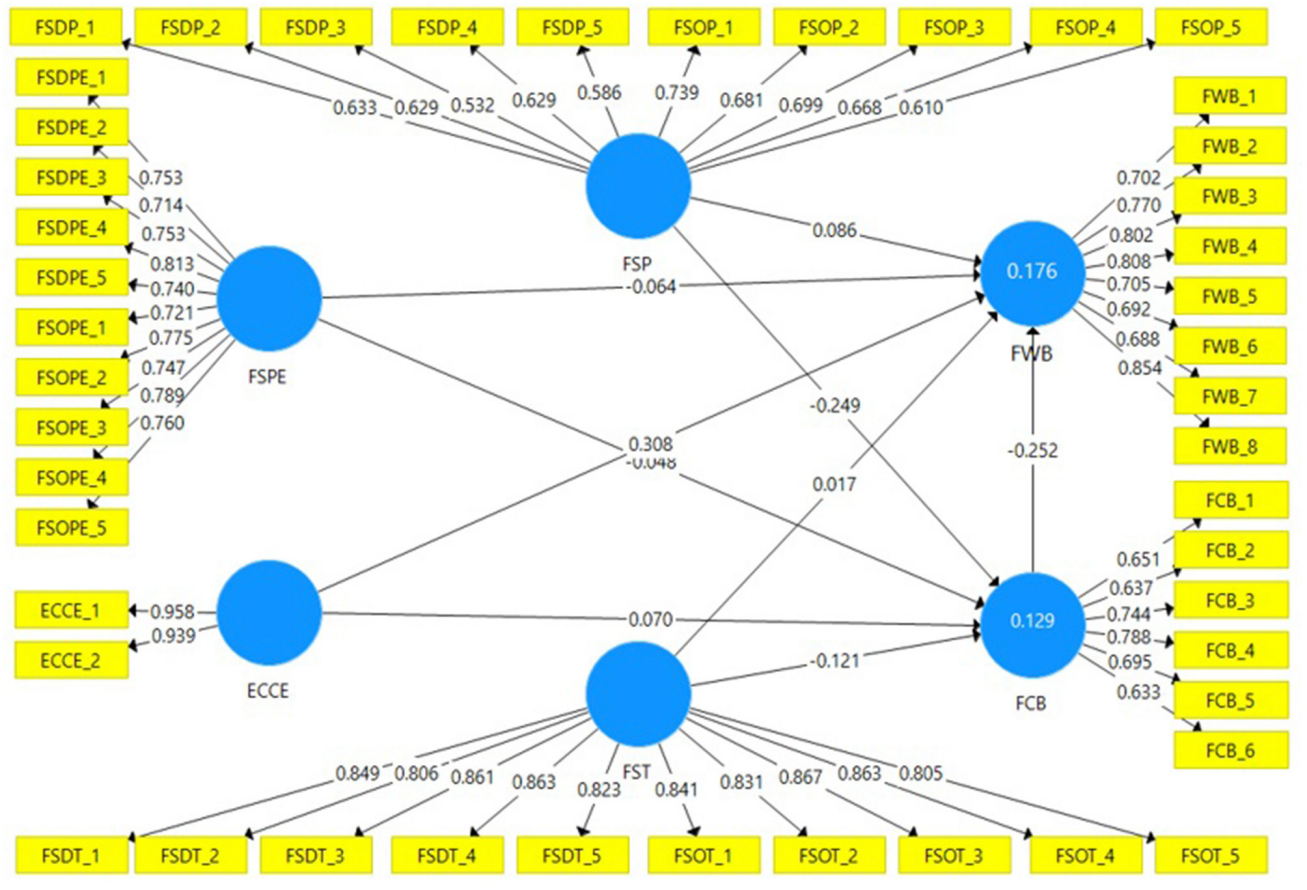
Direct, indirect, and total effect of the relationships. Source: Researcher.

The coefficients was determined by following (Hair et al., 2017). The researchers used the bootstrapping re-sampling technique (5,000 subsamples of the initial sample) to extract the significance values of the indicators. Table 5, Figure 2 show complete projections on the mediation of FWB. Originally, H1 suggested that FSP, FSPE, and FST impact the FWB of adults. Table 5, Figure 2 also show that FSP (*b* = 0.086, *t* = 2.225, *p* < 0.5) has a favorable and statistically meaningful impact on FWB. This result indicates that, with the improvement in the FSP, the FWB will be improved. We will hold our H1a study hypothesis that the FSP has a major impact on the FWB.

**Table 5:** Direct, indirect, and total effect of the relationships.

	**Direct effect**	**Indirect effect^1^**	**Total effect**
ECCE -> FCB	0.07^a^		0.07^a^
	(2.086)		(2.086)
ECCE -> FWB	0.308^b^	−0.018^a^	0.29^b^
	(10.215)	(2.235)	(9.583)
FCB -> FWB	−0.252^b^		−0.252^b^
	(5.96)		(5.96)
FSP -> FCB	0.249^b^		0.249^b^
	(6.945)		(6.945)
FSP -> FWB	0.086^a^	0.063^b^	0.148^b^
	(2.224)	(4.258)	(3.935)
FSPE -> FCB	−0.048		−0.048
	(1.204)		(1.204)
FSPE -> FWB	−0.064	0.012	−0.052
	(1.533)	(1.149)	(1.226)
FST -> FCB	−0.121^b^		−0.121^b^
	(2.938)		(2.938)
FST -> FWB	0.017	0.03	0.048
	(0.47)	(2.568)^b^	(1.242)

1 *Financial Coping Behaviors (FCB) is a mediating variable*.

*Values in the parenthesis are t values*.

a *p < 0.05*.

b *p < 0.001*.

Statics related to FSPE (*b* = −0.064, *t* = 1.533, *p* > 0.1) showed that FSPE has a negative but statistically insignificant effect on the FWB of the adults. The negative sign of the coefficient showed that, as the value of FSPE increases, the value of FWB decreases, but this relationship is statistically insignificant. Based on the results, we can conclude that FSPE does not affect the FWB of adults. Hence, the researcher found no support for H1b.

The results also revealed that FST (*b* = 0.017, *t* = 0.47, *p* > 0.1) shows that FST has a positive but statistically insignificant effect on the adults' FWB in Pakistan. Hence, the researchers found no support for H1c. To sum up all the statistics related to H1c, we can conclude that FS has a partial effect on the FWB, as only FSP was relevant to the FWB. The study results revealed that only socialization with parents significantly affects FWB among all FS agents. Hence, the researcher found partial support for H1c.

H2 was that ECCE affects FWB. Table 5, Figure 2 show that ECCE (*b* = 0.308, *t* = 10.215, *p* < 0.01) has a significant effect on the FWB. This result confirms that with ECCEs, the value of FWB increases. Hence, the results of the study supported H2.

H3 was that FCB mediates the impact of FSP, FSPE, FST, and FWB. Table 5, Figure 2 show the indirect effect from FS to ECCE and FWB, and Table 6 reports the results of the Sobel test and VAF statistics used to test the significance of the mediating variable. The coefficient of indirect effect of FSP is *b* = 0.063, *t* = 4.258, and *p* < 0.01. The statistics related to the indirect effect of FCB between FSP and FWB have a significant indirect effect. FCB's mediation effect coefficient between FSP and FWB is *b* = 3.63 and *p* < 0.001, which confirms that the mediation effect is statistically significant. The analysis of VAF shows that FCB partially mediates the relationship between FSP and FWB. Based on the results, we can conclude that FCB mediates the relationship between FSP and FWB of Pakistan's young adults. Hence, we will accept H3a.

**Table 6:** Mediation analysis.

**Hypothesis**	**Sobel test**	**VAF**	**Mediation**
**H**_**3a**_**:** The FCB mediates the relationship between FSP and FWB.	3.63 (0.000)	42.56%	Partial mediation
**H**_**3b**_**:** FCB mediates the relationship between FSPE and FWB.	0.831 (0.406)	N/A	No mediation
**H**_**3c**_**:** The FCB mediates the relationship between FST and FWB.	1.934 (0.053)	62.5%	Partial mediation
**H**_**4**_**:** FCB mediates the relationship between ECCE and FWB.	1.525 (0.128)	N/A	No mediation

*Values in the parenthesis are P-values*.

*VAF stands for variance accounted for*.

The coefficient of indirect effect of FSPE (*b* = 0.012, *t* = 1.149, *p* > 0.1) shows that FCB has no indirect effect on the relationship between FSPE and FWB. The Sobel test (*b* = 0.831, *p* > 0.1) also confirms that FSPE does not affect FSPE and FWB's relationship. Hence, the researchers found no support for H3b.

For the indirect effect of FCB, the coefficient value (*b* = 0.03, *t* = 2.568, *p* < 0.01) confirms the positive and statistically significant indirect of the FCB on the relationship between FST and FWB. Furthermore, the Sobel test (*b* = 1.934, *p* < 0.1) shows the mediating effect of FCB on FST and FWB's relationship. The analysis of VAF confirms that FCB partially mediates the relationship between FST and FWB. Hence, the researcher accepts H3c. Overall, the present study provided partial support H3.

H4 was that FCB mediates the relationship between ECCE and FWB. The indirect effect from Table 5, Figure 2 reveals that the coefficient of ECCE (*b* = −0.018, *t* = 2.235, *p* < 0.01) confirms the indirect effect of FCB on the relationship between ECCE and FWB. The Sobel test (*b* = 1.525, *p* > 0.1) reveals that the FCB's mediation effect on the relationship between ECCE and FWB is statistically insignificant. Hence, the researchers found no support for H4.

## Discussion and Conclusion

The present research aims to establish and evaluate a conceptual model of FWB while focusing on FS and ECCE. This research is among the few early studies investigating the connection among FS, ECCE, and FWB. The findings of the study provided support to our theoretical model. The theories of FS, lifespan growth, and expected actions formed the foundation for the theoretical model of the analysis.

The present study supported the notation that we can improve the FWB of adults through the FS process and childhood consumer experiences. Contrary to previous studies (Gutter et al., 2010; Mohamed, 2017; Rea, 2017; Abbas et al., 2019b), our study showed that peers' FS has no direct nor indirect effect on the FWB of the individuals. This finding shows that adults do not take the influence of peers while making financial decisions. Usually, people who try to follow and observe their peers' financial patterns often face difficulties managing their financials. These difficulties arise because of the lack of confidence and lack of peer support at the time of stress. Thus, individuals in Pakistan do not consider peers as socialization agents. Surprisingly, individuals also do not consider teachers as socialization agents. Drever et al. (2015) provided support for FST in explaining positive financial behaviors in adults. However, children in Pakistan do not discuss or observe the financial behaviors of their teachers. The descriptive statistics analysis also reveals the same thing that individuals do not observe, discuss, and follow their teachers' financial behaviors. One possible explanation for this event is that, in many countries, including America and China, teachers teach financial concepts to school-going children. When the teachers teach them, students can take their influence in making financial decisions. However, in Pakistan, no concept of teaching finance-related subjects to young students exists. Normally, students start learning basic concepts of finance after grade 12. Owing to this curriculum difference, teachers do not affect the adults' FWB in Pakistan.

Consistent with the previous studies, our study supported that parents' FS has direct and indirect effects on the adults' FWB. The study results are consistent with (Sabri, 2011; Kim and Chatterjee, 2013; Drever et al., 2015; Rea, 2017; Rea et al., 2019). They also argued that FSP has a positive impact on FWB. The results confirm that parents are considered important FS agents, and their interaction with the child helps manage financials properly, resulting in FWB.

ECCE has a clear impact on adult FWB. Consistent with Rea et al. (2019), Sabri et al. (2012), this research confirms the assumption that adults with bank accounts or other childhood investment strategies have a positive effect on adult financial decisions. ECCE offers adults trust that they can handle their money properly. Proper financial control aims to achieve FWB through adulthood. Peng et al. (2007) claimed that practice allows to make sound investments and save money than financial education.

The role of copying behaviors is also essential in achieving FWB among adults. This role gives them inner motivation to work hard and find a solution to financial problems. CB also helps individuals in lowering distress related to financial decisions. The study results showed that CBs associated with early experiences and FS help individuals achieve FWB by motivating them to work hard to achieve FWB. However, FCB worked as a mediator only for FSP and FST. Earlier, only Serido et al. (2010) studied the mediating role of CBs in explaining the relationship between FSP and FWB and concluded that FCB works well as a mediator between FSP and FWB. The present study results are in line with Serido et al. (2010). That is, adults develop CBs that improve their FWB through the interaction between parents and children in their childhood. No particular study considered the mediating role of FCB in the relationship between FST and FWB. However, the results of the present study showed that FCB mediates the relationship between FST and FWB. This finding confirms that children with the interaction with teacher only develops the coping skills that result in improved FWB.

Individuals' financial decisions are likely to be affected by those with whom they associate. People in their adulthood typically spend more time with their parents and obey their decision-making habits. This claim was supported by the results of the study. This result indicates that parents are in a stronger place to advise adults on financial choices. The previous literature also supported our study (Oaten and Cheng, 2007; Bucciol and Veronesi, 2014). The result of the present study failed to provide evidence that FSPE and FST affect FWB. Thus, adults do not consider the role of teachers and peers as socialization agents. The present study supports that adults with ECCEs positively affect adulthood's financial decisions. ECCE offers adults encouragement that they can handle their financial resources. Discussion with parents enables self-regulation abilities in individuals that enhance coping actions in individuals. This event helps adults to face financial challenges more easily because of their faith in addressing and observing the financial problems of their parents. In other terms, owing to self-confidence, adults can deal with the financial challenges they encounter and maintain FWB. The findings indicated that individuals who discuss and follow the financial decision-making habits of their parents and have coping mechanisms should boost their FWB.

The empirical results highlighted the positive role of parents in improving FWB among adults. Whether parents transfer their skills and knowledge to their children to manage financials in the future is under debate. However, the present study results pointed out parents' direct effect on the adults' FWB. One possible explanation for this case is the lack of literacy among the parents. Approximately 43% of fathers and 60% of mothers have not attended university. As in Pakistan, the financial literacy situation was poorer as most institutions started financial literacy programs after the 1990s. Hence, parents are unable to influence FWB among adults directly. The problem of the lack of financial literacy can be solved by educating parents. The educational programs for parents will provide the skills and knowledge required to educate their children. When parents have the skills and knowledge to manage financials, they can properly guide their children to manage financials and achieve FWB. Offering informal financial education can be a good idea to promote financial literacy in adults, which can be achieved by organizing workshops and seminars related to money management skills. Counseling can also be helpful to the parents in acquiring financial skills and knowledge. In sum, parents should attend, and policymakers should arrange literacy programs where parents can improve their knowledge and skills.

The direct role of ECCEs is also highlighted in the study. Children can only be involved in ECCEs if their parents allow them. Thus, ECCEs are also related to the parents. Children who practice financial management skills in their childhood are believed to perform better in their adulthood. When children have the opportunity to manage their pocket money themselves, they try to learn and follow money management skills that improve their confidence, skills, and most importantly, their FWB. LeBaron et al. (2018) provided examples of money management skills, such as opening a bank account, recording expenses, keeping a record of shopping, observing, and discussing parents and siblings. However, in Pakistan, the situation is different. Although such management skills showed a significant effect, ECCE's average score showed that most individuals start practice money management skills after reaching 25 years. Children are not encouraged to make financial decisions independently from their parents. The role of the government is also not supportive. No mechanisms exist through which children can open their bank accounts and manage their financials. Parents should keep in mind the importance of ECCEs and provide opportunities for their children to practice money management skills in their childhood.

Moreover, policymakers should work on how children can start managing their finances at an early age. That can be done by training parents and children and restricting them to open saving and investing mechanisms. In China and America, many institutes frequency visits in the schools guide money management skills, and in Pakistan Chinese experience can be utilized.

Despite being a comprehensive study, the current study has significant limitations. Self-administered questionnaires were utilized in this study to collect data from the public based on their opinions. Individuals responded to certain questions based on their memories. To obtain more reliable data, many sources of information should be used. We cannot fully investigate the causal link among variables using cross-sectional data. Longitudinal data are needed to test the causal relationship. However, collecting longitudinal data quickly was not possible, so the researcher only used cross-sectional data. In some recent related studies, the role of religion, financial literacy, and parents' social setup is also studied. These should be considered in future studies. In explaining FWB in more detail, the role of some other financial behaviors should be studied along with these variables. Media played an important role in shaping individuals' decisions. The researchers also recommend studying the role of media as a socialization agent. In the present study, the researcher tried to explore this but was limited by the non-availability of instruments to measure the media's role as a socialization agent. During the data collection, some experts argued that the roles of siblings and spouses could also be important; however, as data collection was made, contacting the respondents again to obtain their response related to spouse and siblings as FS agents was impossible. In the present study, the researchers collected data from Pakistan only. Pakistan is a developing country, and its population faces certain problems, but many other countries do not face these problems. In Pakistan, the children are mostly attached to their parents, and they prefer to live with them and support each other. In western countries, governments take responsibility for taking care of the children, whereas in Pakistan, parents perform these types of duties. Hence, the results of the present study are specific to Pakistan only.

As the coronavirus pandemic spreads rapidly throughout the world, it is instilling widespread fear, worry, and concern in the general population and in particular among certain groups, such as older adults, caregivers, and people with underlying health conditions and well-being (Abbas, 2020; Aman et al., 2021; Aqeel et al., 2021; Maqsood et al., 2021). Therefore, in this era, it is more important to study financial well-being and its related factors.

We are living in an ever-moving world where everything is changing. Similarly, savings, investments, and money management beliefs and products are also changing. Moreover, there no products such as derivate securities exist in the financial markets. However, now, these products are a reality and are traded on the stock markets. Similarly, no cryptocurrency concept existed just 15 years ago, but now, many countries are trying to move toward cryptocurrencies. New generations are more used to these products. Instead, parent's guide their children; now, children can guide their parents about these products so that this parent–children role can be bidirectional. Therefore, considering this bidirectional role, future studies should be conducted. The role of media, siblings, and spouse as a socialization agent can also be critical. In the present study, these socialization agents were ignored, but scholars should consider these in future studies. Similarly, cultural issues and religious aspects are also ignored, which should also be considered in future studies. Longitudinal data can provide more insights into these relationships. The use of longitudinal data can provide insights into who and why individuals' perception changes over time—the present study, owing to time and financial constraints, only used cross-sectional data. In the future, researchers should consider longitudinal data for similar studies. Demographic aspects can also be important. In the present study, the researchers collected data only from Pakistan, where most of the respondents had similar characteristics. The present model should be applied in different developing countries and compare their findings to obtain more insights into the results.

## Data Availability Statement

The raw data supporting the conclusions of this article will be made available by the authors, without undue reservation.

## Ethics Statement

This study was carried out in accordance with the recommendations of the Ethical Principles of Psychologists and Code of Conduct by the American Psychological Association (APA). The participants provided their written, informed consent to participate in this study.

## Author Contributions

JS provided technical assistance, actively participated in all the steps followed in this study, helped in conceptualization, and improving this draft. SU was involved in all the steps and procedures followed in this study, conceptualization, reviewing the literature, finalizing research methodology, data collection and analysis, and writing and reviewing the original draft. XZ, SD, and FS played an important role in facilitating data collection, completing this project, most importantly, and in improving the quality of the present draft. All authors contributed to the article and approved the submitted version.

## Funding

The authors of the present study admiringly acknowledge the financial support of the National Natural Science Funding (Grant Number: 71904065) and the High Talent Research Program by Jiangsu University (17JDG005).

## Conflict of Interest

The authors declare that the research was conducted in the absence of any commercial or financial relationships that could be construed as a potential conflict of interest.

## Publisher's Note

All claims expressed in this article are solely those of the authors and do not necessarily represent those of their affiliated organizations, or those of the publisher, the editors and the reviewers. Any product that may be evaluated in this article, or claim that may be made by its manufacturer, is not guaranteed or endorsed by the publisher.
